# Lunar life drives jawbone formation

**DOI:** 10.1016/j.jds.2025.03.032

**Published:** 2025-04-09

**Authors:** Kotaro Sato, Naoka Kouyama, Shinya Akatsuka, Yashiro Motooka, Qinhong Wang, Hideharu Hibi, Shinya Toyokuni

**Affiliations:** a Department of Oral and Maxillofacial Surgery, Nagoya University Graduate School of Medicine, Nagoya, Japan; b Department of Pathology and Biological Responses, Nagoya University Graduate School of Medicine, Nagoya, Japan; c Center for Low-Temperature Plasma Sciences, Nagoya University, Nagoya, Japan; d Center for Integrated Sciences of Low-temperature Plasma Core Research (iPlasma Core), Tokai National Higher Education and Research System, Nagoya, Japan

**Keywords:** DMP1, Hedgehog signaling, Jawbone, Lunar gravity, Mastication, SPP1

## Abstract

**Background/purpose:**

The Japan Aerospace Exploration Agency bred mice in the “Kibo” module on the International Space Station to clarify the effects of gravity changes on the body. We determined the effects of lunar gravity (partial gravity, 1/6G) on the jawbone and elucidated its relationship with the oral cavity.

**Materials and methods:**

Mandibular bones of C57BL/6J male mice from the Kibo group (1/6G) and the Earth group (1G) were obtained. Ribonucleic acid was extracted from the samples; microarray and gene set enrichment analysis (GSEA) were performed; bone structure and histology were evaluated via micro-computed tomography, immunohistochemistry, and specific staining.

**Results:**

Microarray analysis revealed a significant increase in dentin matrix protein 1 (DMP1) expression in the 1/6G group. GSEA revealed enhanced Hedgehog signaling in this group. The weight and density of the cancellous bone were significantly greater in the 1/6G group than in the 1G group, as confirmed by histological analysis. Furthermore, the expression of insulin-like growth factor 1 (IGF-1), which is associated with mastication-induced bone formation, was increased in the 1/6G group. Although secreted phosphoprotein 1 (SPP1), which is associated with bone resorption under low mechanical stress, increased in the 1/6G group, receptor activator of nuclear factor kappa-B ligand (RANKL)-RANK signaling did not increase. No significant differences in the number or activity of osteoclasts were observed between the 1G and 1/6G groups.

**Conclusion:**

Bone formation, but not bone resorption, is promoted by lunar gravity and is associated with mastication. Proper oral management is necessary for extended lunar habitation.

## Introduction

Gravity on Earth results from terrestrial gravitation and the Coriolis force and is inseparably linked to our lives; this force is often taken for granted. Understanding the role of gravity in life will help elucidate the basic physiological mechanisms involved in the differentiation, development, and evolution of animals, plants, and humans. However, extraterrestrial colonization may eventually become necessary for the survival of the human species.

The Artemis mission, a 2024 human lunar exploration plan by the National Aeronautics and Space Administration (NASA), aims to send humans to the Moon after 2025, transport supplies through the Gateway (a manned lunar orbit base) project and construct a lunar base to sustain human activities. The space environment is believed to accelerate biological changes such as bone loss, muscle atrophy, inner ear dysfunction, and immune dysfunction, similar to those observed in older individuals who are bedridden on Earth.^1^ Kibo is an experimental module developed in Japan and the country's first manned facility where astronauts can operate for long periods. It is also the largest module on the International Space Station (ISS). Kibo has conducted microbiological research using various model organisms to clarify the mechanisms of aging-like phenomena on Earth and establish countermeasures. Although some studies have reported the weakening of limb muscles and bones due to microgravity,^2^^,^^3^ no studies have examined changes in the oral cavity, which plays an essential role in feeding. For future sustainable activities on the Moon, understanding the effects of low gravity on the oral cavity, particularly the jawbone, is essential, as eating is essential for survival in a low-gravity environment.

We aimed to investigate the effects of low gravity in the space environment on the jawbone and clarify the relationship between space and the oral cavity, particularly the jawbone.

## Materials and methods

### Animals

**Figure 1:**
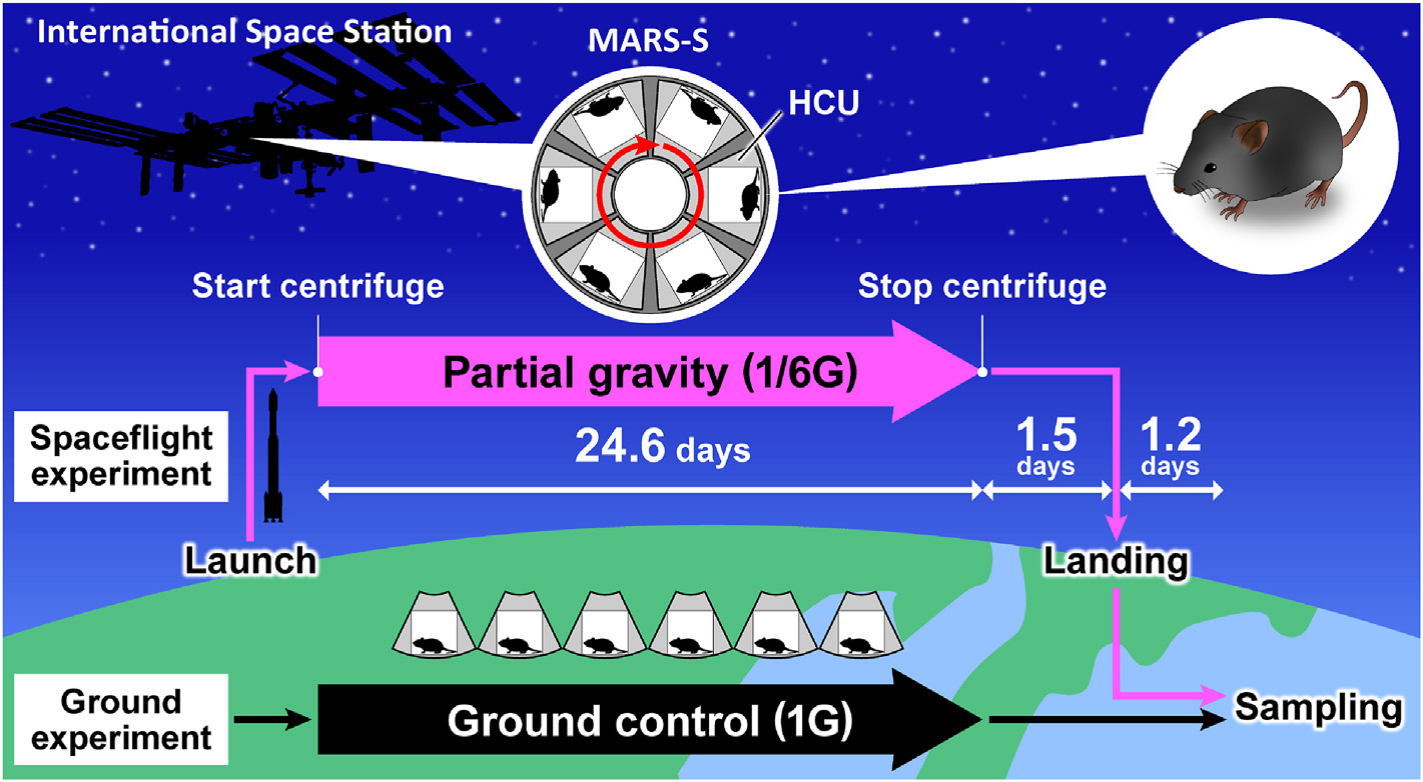
**Schematic representation of flight conditions and the experiment**. C57BL/6J male mice (9 weeks old) were obtained from the Kennedy Space Center via a transportation cage unit (TCU). On the International Space Station (ISS), the mice were transferred into habitat cage units (HCUs) installed in the Multiple artificial gravity research system-short (MARS-S) for gravity loading. Gravity was set to 1/6G (0.167 g = partial gravity = lunar gravity) to mimic the gravity of the Moon. In the 1G group, the mice were housed on Earth in the same MHU as those in the 1/6G group. After being housed onboard for a certain period, the mice were transferred back to the TCU and landed on the west coast of the USA.

C57BL/6J male mice (stock #000664) were purchased from Jackson Laboratories (Bar Harbour, ME, USA) and Charles River Laboratories (Yokohama, Japan). This experiment was approved by the Institutional Animal Care and Use Committee of the Japan Aerospace Exploration Agency (JAXA) (protocol number: No. 018-011D), Explora Biolabs (study number: No. EB19-003), and NASA (protocol number: No. FLT-18-118). All experiments were conducted in accordance with the guidelines and applicable laws in Japan and the USA. Six mice in the transportation cage unit were launched aboard SpX-17 on May 7, 2019, from NASA's Kennedy Space Center and transported to the ISS. To determine the effects of different gravitational forces on mice during spaceflight, they were maintained on the ISS for approximately 1 month (Fig. 1). Gravity was set to 1/6G (0.167 g = lunar gravity = partial gravity) to mimic the gravity of the Moon, as its exploration is the most feasible future mission. Habitat cage units were placed on the floor, each housing six mice, and a short-radius centrifuge (MARS-S) generated 1/6G. Conversely, mice in the 1G group were housed on Earth in the same mouse habitat unit as those in the 1/6G group. Both groups of mice were administered CRF-1 during the experiment. Detailed information, including environmental parameters and habitat data, has been summarized in previous reports.^2^^,^^3^ All mice in the 1/6G group were euthanized on the second day after returning to Earth.

### Ribonucleic acid (RNA) extraction

The harvested tissues were fixed in 4 % phosphate-buffered formalin for 24–48 h at 4 °C. After fixation, the tissues were placed in phosphate-buffered saline (PBS) for approximately 40 days, and PBS was sequentially replaced with 25, 50, 75, or 100 % methanol. RNA was extracted from the samples using a miRNeasy® FFPE Kit (QIAGEN, #217504, Hilden, Germany), RNase-Free DNase Set (QIAGEN, #79254), NanoDrop™ One (Thermo Fisher Scientific, Waltham, MA, USA), and Agilent RNA 6000 Nano Kit (Agilent Technologies, Inc., #5067-1511, Santa Clara, CA, USA). The quality was assessed using an Agilent 2100 Bioanalyzer (Agilent Technologies, Inc.).

### Microarray and gene set enrichment analysis (GSEA)

We used a Clariom™ S Assay Mouse (Thermo Fisher Scientific, # 902,930) for this assay. First, we produced fragmented and labeled complementary deoxyribonucleic acid (cDNA) from 50 ng of total RNA with a GeneChip™ 3′ IVT Pico Kit (Thermo Fisher Scientific, # 902,789) and prepared 100 μL of hybridization mixture (73 μL of hybridization master mix + 5.0 μg of fragmented and labeled cDNA). The arrays were incubated for 16 h (60 rpm) at 45 °C in a GeneChip™ Hybridization Oven 645 (Thermo Fisher Scientific) and washed with a GeneChip™ Fluidics Station 450 (Thermo Fisher Scientific) according to the manufacturer's protocol. The samples were scanned using a GeneChip Scanner 3000 7G (Thermo Fisher Scientific). We analyzed the results using the Transcriptome Analysis Console (Thermo Fisher Scientific). Hierarchical clustering analysis was also performed on the expression profiles of the 63 differentially expressed genes. We normalized the profile data of each gene via z-score transformation and used Ward's clustering method for analysis. Based on the microarray data, we performed GSEA (http://software.broadinstitute.org/gsea/) to confirm the pathway changes. Microarray data were deposited in the GEO repository (accession number: GSE286071).

### Evaluation of mandibular bone quality via computed tomography (CT)

Mandibular bones were imaged with a Cosmo Scan GXII (Rigaku, Tokyo, Japan) using the following camera settings: exposure time, 4 min; field of view, 18 mm. We measured the average tissue volume, bone volume, bone surface area, and bone volume fraction (bone volume/tissue volume) from the distal terminal molar to the mandibular ramus, excluding the teeth. Data were analyzed via the bone analysis software TRI/3D-BON (RATOC, Tokyo, Japan).

### Histological analysis

Fixed tissues were decalcified with 10% ethylenediaminetetraacetic acid disodium salt, embedded in paraffin, sectioned at 5 μm, mounted on glass slides, and deparaffinized before hematoxylin and eosin and picrosirius red staining. To analyze the distribution of osteoblasts and osteoclasts, we used a tartrate-resistant acid phosphatase/alkaline phosphatase staining kit (Wako, # 294–67001, Osaka, Japan) according to the manufacturer's protocol. Immunohistochemistry (IHC) was performed on a BOND-MAX system (Leica Microsystems GmbH, Wetzlar, Germany) using the manufacturer's reagents following the manufacturer's protocol. Anti-dentin matrix protein 1 (DMP1) (Invitrogen, PA5-103323, Carlsbad, CA, USA), secreted phosphoprotein 1 (SPP1) (Invitrogen, PA5-141,129), runt-related transcription factor 2 (RUNX2) (Abcam, ab192256, Cambridge, UK), bone morphogenetic protein 2 (BMP2) (Bioss, bs-1012R, Boston, MA, USA), Sp7 (Abcam, ab209484), cathepsin K (Abcam, ab300569), and insulin-like growth factor 1 (IGF-1) (Proteintech, 28530-1-AP, Rosemont, IL, USA) antibodies were used after dilution with primary antibody diluent (Leica). For the IGF-1 intensity score, we set four scores (0–3) for the osteocytes and randomly selected fields. All osteocytes were scored, and the average intensity was calculated.

### Statistical analysis

Statistical analyses were performed using Student's t-test with GraphPad Prism 9 (GraphPad Software, San Diego, CA, USA). *P* < 0.05 indicated significance. Data are presented as the mean ± standard error of the mean.

## Results

### Life in lunar gravity (1/6G) upregulates DMP1 and SPP1 and promotes the bone maturation pathway

**Figure 2:**
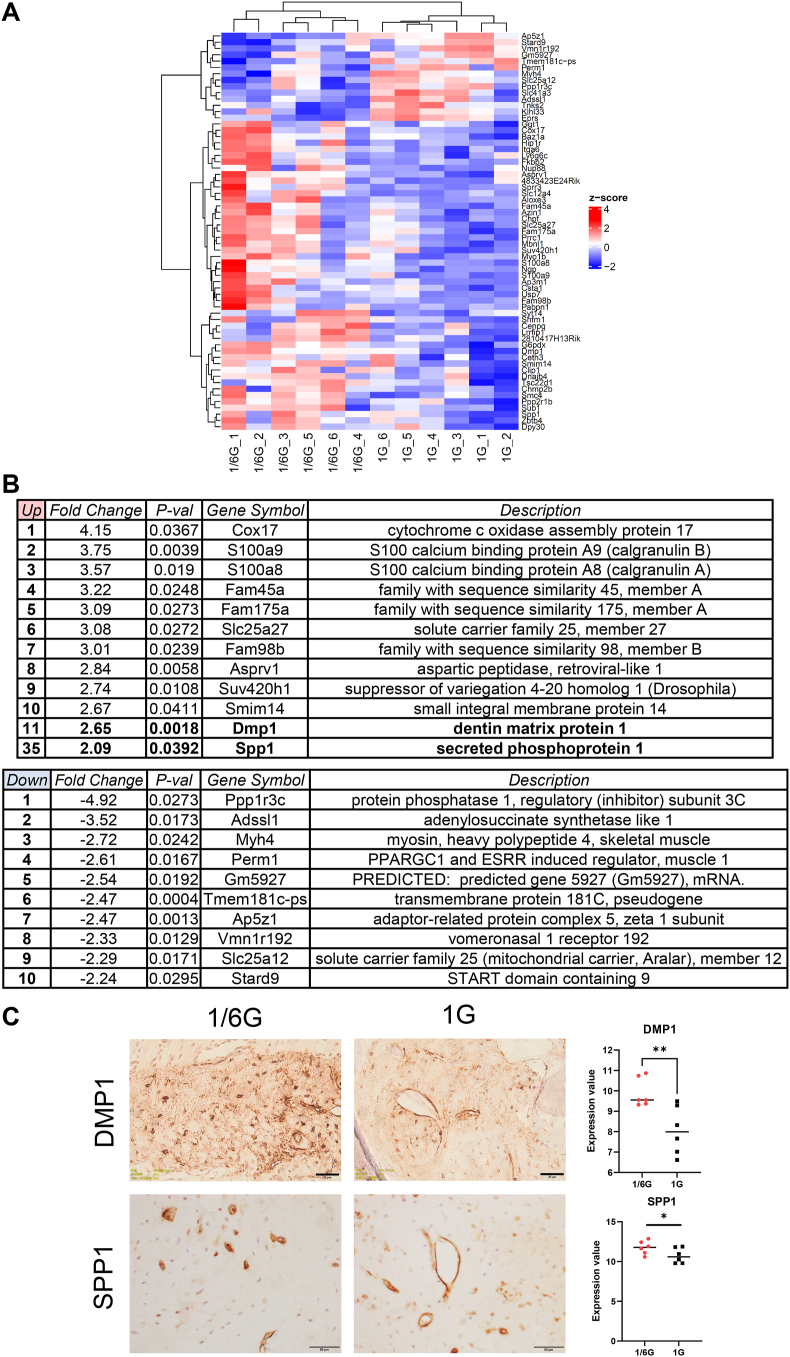
**Analysis of gene expression profiles under partial gravity (PG: 1/6G) and ground control (GC: 1G) conditions**. (A) Clustering heatmap of significantly differentially expressed genes between the 1/6G and 1G groups. (B) Upregulated and downregulated genes. (C) Histological findings and gene expression of dentin matrix acidic phosphoprotein 1 (DMP1) (scale bar = 20 μm) and secreted phosphoprotein 1 (SPP1) (scale bar = 50 μm) between 1/6G and 1G. ∗*P* < 0.05 and ∗∗*P* < 0.01.

We performed a microarray analysis to determine the effects of lunar gravity on the jawbone in the space environment. DMP1 expression significantly increased in the 1/6G group (Fig. 2A and B), and GSEA revealed an increase in Hedgehog (Hh) and transforming growth factor beta (TGF-β) signaling in the same group (Supplementary Fig. 1A). Furthermore, SPP1 expression was upregulated at 1/6G, and similar results were obtained using immunohistochemical staining (Fig. 2B and C). Although RUNX2, BMP2, and Sp7 levels were slightly increased in the 1/6G group, no significant differences were observed, similar to the IHC results (Supplementary Fig. 1B).

### IGF-1 expression is increased, as is type I collagen expression, leading to an increase in bone density during lunar gravity

**Figure 3:**
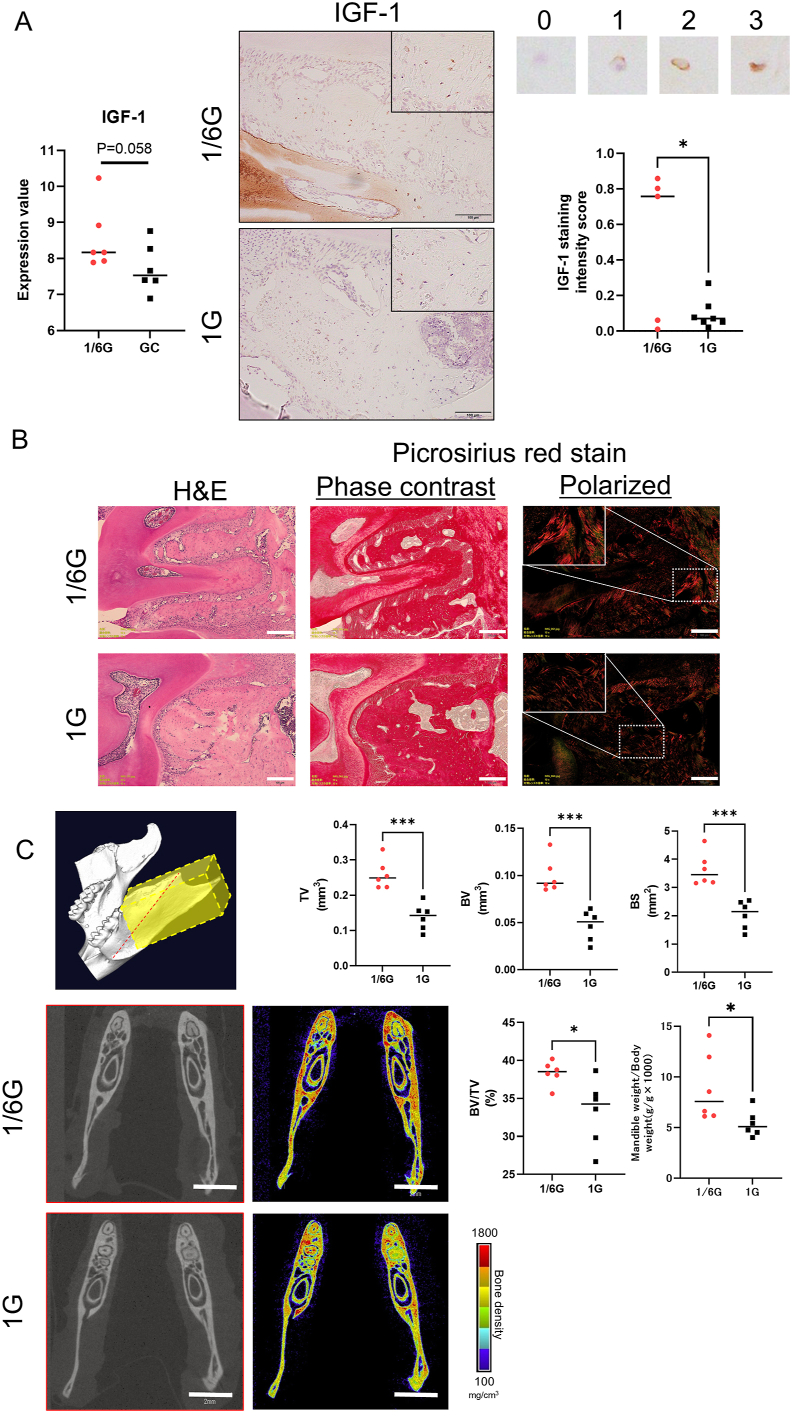
**Effect of partial gravity on the mandibular bone**. (A) Histological findings and gene expression of insulin-like growth factor 1 (IGF-1) between 1/6G and 1G. The intensities were categorized into 4 groups (0–3) and randomly selected some fields. Scale bar = 100 μm. (B) Histological findings of type Ⅰ collagen at 1/6G and 1G. Scale bar = 100 μm. (C) Quantification of mandibular bone using CT images between 1/6G and 1G. Scale bar = 2 mm. Yellow box: measuring area; red dotted line: cutting plane of the images. ∗*P* < 0.05 and ∗∗∗*P* < 0.001. ns, Not significant. TV: Tissue volume, BV: Bone volume, BS: Bone surface, BV/TV: Bone volume fraction.

We investigated the role of mastication in bone formation. IHC revealed a significant increase in IGF-1 expression in osteocytes at 1/6G (*P* = 0.058; Fig. 3A). Picrosirius red staining revealed increased brightness at 1/6G (Fig. 3B). The mandibular weight-to-body weight ratio was greater in the 1/6G group than in the 1G group. Micro-CT revealed significant increases in bone volume fraction, tissue volume, bone volume, and bone surface area (Fig. 3C).

### Lunar gravity did not change the number or activity of osteoclasts or promote receptor activator of nuclear factor kappa-B ligand (RANKL)-RANK signaling

**Figure 4:**
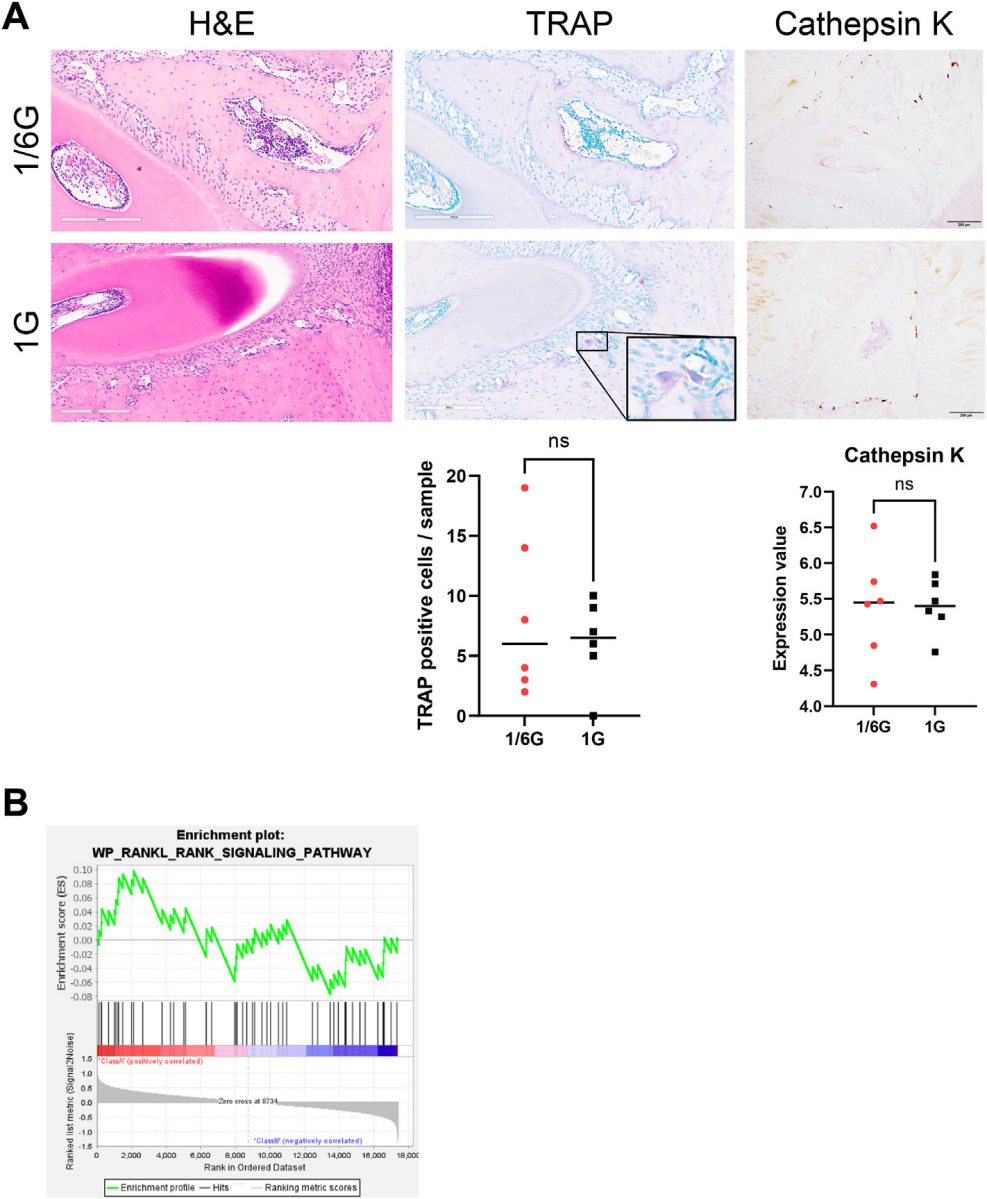
**Bone resorption factors in the mandibular bone**. (A) Number and activity of osteoclasts in the 1/6G and 1G groups. (B) receptor activator of nuclear factor-kappa B ligand (RANKL)-RANK signaling from gene set enrichment analysis (GSEA). Scale bar = 200 μm. TRAP: Tartrate-resistant acid phosphatase. ns, Not significant.

Tartrate-resistant acid phosphatase staining showed no increase in the number of osteoclasts in the 1/6G group. Furthermore, no significant difference was observed in cathepsin K expression at either the RNA or IHC levels (Fig. 4A). GSEA showed no promotion of RANKL-RANK signaling (Fig. 4B).

## Discussion

**Figure 5:**
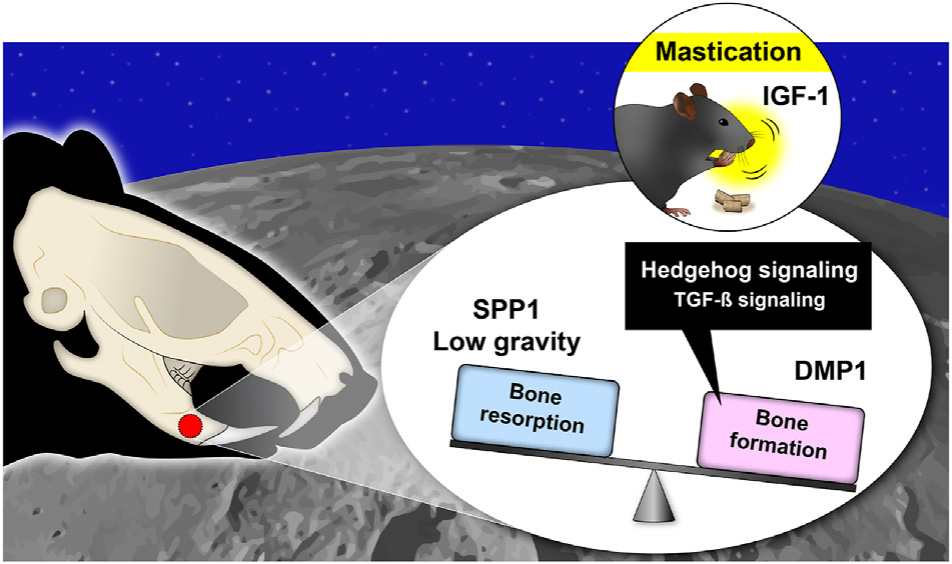
**Overview of the effect of lunar gravity on the jawbone**. Bone formation was promoted, while resorption was not, under lunar gravity (1/6G), suggesting an association with mastication. DMP1: Dentin matrix acidic phosphoprotein 1, IGF-1: Insulin-like growth factor 1, SPP1: Secreted phosphoprotein 1.

Under lunar gravity (1/6G), bone formation markers in the mandible are upregulated, and bone density increases, which might be related to mastication. Surprisingly, no bone resorption was observed. These results suggest that when people live and eat the same food as they do on Earth under lunar gravity, bone formation, rather than resorption, is promoted in the jawbone (Fig. 5). Although atrophy of the bones and muscles of the limbs is known to occur under lunar gravity,^2^^,^^3^ the opposite phenomenon was observed in the jawbone in this study.

First, a comprehensive analysis was conducted to clarify the changes that occurred in the samples. We found that some factors related to bone metabolism changed and focused on these factors to advance this research. DMP1 is a non-collagenous protein expressed in bones and teeth and is specifically expressed in osteocytes in bone tissue.^4^^,^^5^ This protein is involved in calcification in bone, and when DMP1 is suppressed, fibroblast growth factor 23 (FGF23) is highly expressed in bone cells; FGF23 in the blood can increase, causing autosomal recessive hypophosphatemic rickets.^6^ Therefore, DMP1 suppresses FGF23 production upstream of bone cells, controls phosphorus metabolism, and participates in bone cell maturation. In this study, DMP1 expression in osteocytes increased in the Kibo group (2.65 times, *P* = 0.0018), and bone density also increased in the CT images, indicating that bone formation is promoted in a low-gravity environment.

In general, there are two types of ossification patterns: intramembranous and endochondral. In intramembranous ossification, aggregated mesenchymal cells differentiate into osteoblasts, and bone formation progresses, whereas, in endochondral ossification, mesenchymal cells differentiate into chondrocytes and osteoblasts, with bone formation occurring after cartilage formation. This pathway involves quiescent chondrocytes, proliferating chondrocytes, hypertrophic chondrocytes, and osteoblasts. Chondrocytes grow from quiescent to proliferating state while secreting cartilage matrix proteins, such as glycosaminoglycans and collagen types 2, 9, and 10. Eventually, these cells stop proliferating and differentiate into hypertrophic chondrocytes, which secrete type 10 collagen, matrix metalloproteinase-13, and vascular endothelial growth factor. During this process, SRY-box9 acts as the master regulator, whereas RUNX2 and myocyte-specific enhancer factor 2c promote hypertrophic differentiation. Hypertrophic chondrocytes promote surrounding mineralization and angiogenesis. As cartilage is resorbed, a primary ossification center forms and is replaced by bone. Mesenchymal cells in the perichondrium surrounding the cartilage contain osteoblast precursor cells. Some of these cells enter the cartilage interior during angiogenesis and become osteoblasts, thus contributing to the formation of primary ossification centers. Moreover, cells that differentiate into osteoblasts in the periphery form a bone shell, which becomes the source of cortical bone. During osteoblast differentiation, mesenchymal cells first become RUNX2-positive osteoblast precursor cells and then differentiate into osteoblasts expressing Sp7. These cells are the master regulators of osteoblast differentiation. Subsequently, osteoblasts secrete bone matrix proteins such as SPP1, Ibsp, and Bglap, along with type I collagen, to induce matrix mineralization. BMP2 belongs to the TGF-β superfamily and is known as a bone formation factor that induces bone formation.^7^ In this study, RUNX2, Sp7, and BMP2 levels tended to increase, but not significantly, whereas TGF-β signaling was significantly promoted. These findings suggest that bone development may be accelerated under low-gravity conditions in daily life.

Consequently, we examined other factors related to bone formation, including collagen and signaling pathways involved in bone metabolism. Collagen is secreted by the osteoblasts and contributes to osteoid formation and bone calcification.^8^ Histologically, type I collagen is increased, and Hh signaling is promoted, indicating the involvement of multiple factors in bone differentiation. Hh signaling plays a role in coupling chondrogenesis and osteogenesis in endochondral ossification and is an important pathway for the normal differentiation and proliferation of growth plate chondrocytes and the formation of osteoblasts.^9^

SPP1, which is related to bone resorption under low mechanical stress, increased in response to 1/6G. SPP1 is thought to react to mechanical stress, such as tail suspension, and to inhibit bone formation and promote bone resorption.^10^, ^11^, ^12^ This protein constitutes approximately 10 % of the non-collagenous proteins in bone and is relatively abundant in the bone matrix. SPP1 has a gene structure similar to that of DMP1 and belongs to a family of small integrin-binding ligands called N-linked glycoproteins. This protein has various functions in different processes, including bone resorption, inflammation, and bone metastasis in cancer.^13^ Some researchers have reported its effects on disuse bone atrophy in a load-reducing experiment involving tail suspension.^10^, ^11^, ^12^ These studies revealed that bone mass and bone formation decreased while the number of osteoclasts increased in wild-type mice; however, SPP1-deficient mice did not exhibit these changes. Based on these findings, we infer that SPP1 promotes bone resorption under reduced mechanical stress at 1/6G. We also examined the signaling pathways and cells involved in bone resorption. RANKL-RANK signaling was not promoted, and neither the number of osteoclasts nor the level of cathepsin K increased, indicating that osteoclast activity did not increase in the 1/6G group. These results did not indicate the promotion of bone resorption.

To clarify these mechanisms, we focused on mastication as a factor promoting bone formation under 1/6G. The relationship between mastication and jaw development has long been known, as jaw development changes depending on the hardness of the food.^14^ IGF-1 has been reported to be involved in this process.^15^ IGF-1, a growth factor produced by osteoblasts, accumulates in the bone matrix and promotes the proliferation and differentiation of chondrocytes and osteoblasts, as well as matrix synthesis. IGF-1 binds to the IGF receptor and phosphorylates insulin receptor substrate 1,2 (IRS-1, 2), resulting in signal transduction. IRS-1 maintains bone turnover, while IRS-2 promotes bone formation and inhibits bone resorption.^16^^,^^17^ These functions are known to promote bone formation without altering the bone resorption pathway. In this study, IGF-1 levels were significantly increased in the 1/6G group in addition to increased bone density. Although gravity decreases, the hardness of objects does not change under low gravity. Chewing an object with the same hardness as on Earth may require greater bite force under low gravity. The resulting increase in the training effect likely contributed to bone development. However, this may cause an imbalance between the jawbone and teeth, resulting in medical problems such as tooth loosening and jawbone pain. Furthermore, alterations in physiognomy may occur; however, these remained hypothetical. Additionally, because the study duration was short (25 days), the long-term effects of a low-gravity environment remain unclear. Further experimental and hands-on spatial studies are needed to resolve these issues.

When living in space with lunar gravity, unlike on Earth, jawbone resorption is not promoted; instead, bone formation is enhanced, suggesting that mastication may play a role in this process. We believe this research will lead to rapid developments in space stomatology and serve as an important step toward space exploration and future space colonization.

## Declaration of competing interest

The authors declare that they have no known competing financial interests or personal relationships that could have appeared to influence the work reported in this paper.
