# Supplementary material for: Lunar life drives jawbone formation
Source: J Dent Sci. 2025 Apr 9;20(4):2399–406. doi: 10.1016/j.jds.2025.03.032 (PMC12485402; doi:10.1016/j.jds.2025.03.032)
Supplement: Multimedia component 1 [file mmc1.docx]

**Supplementary Material**

**For**

**Lunar life drives jawbone formation**


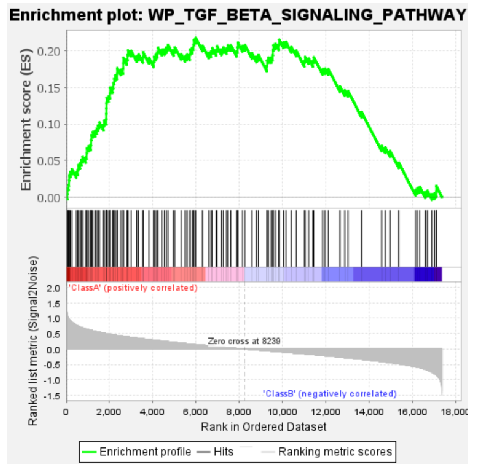
**
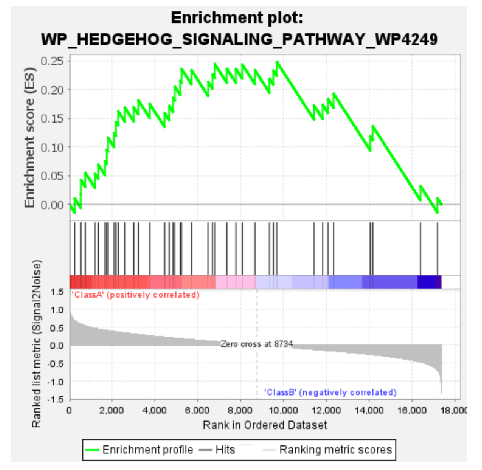

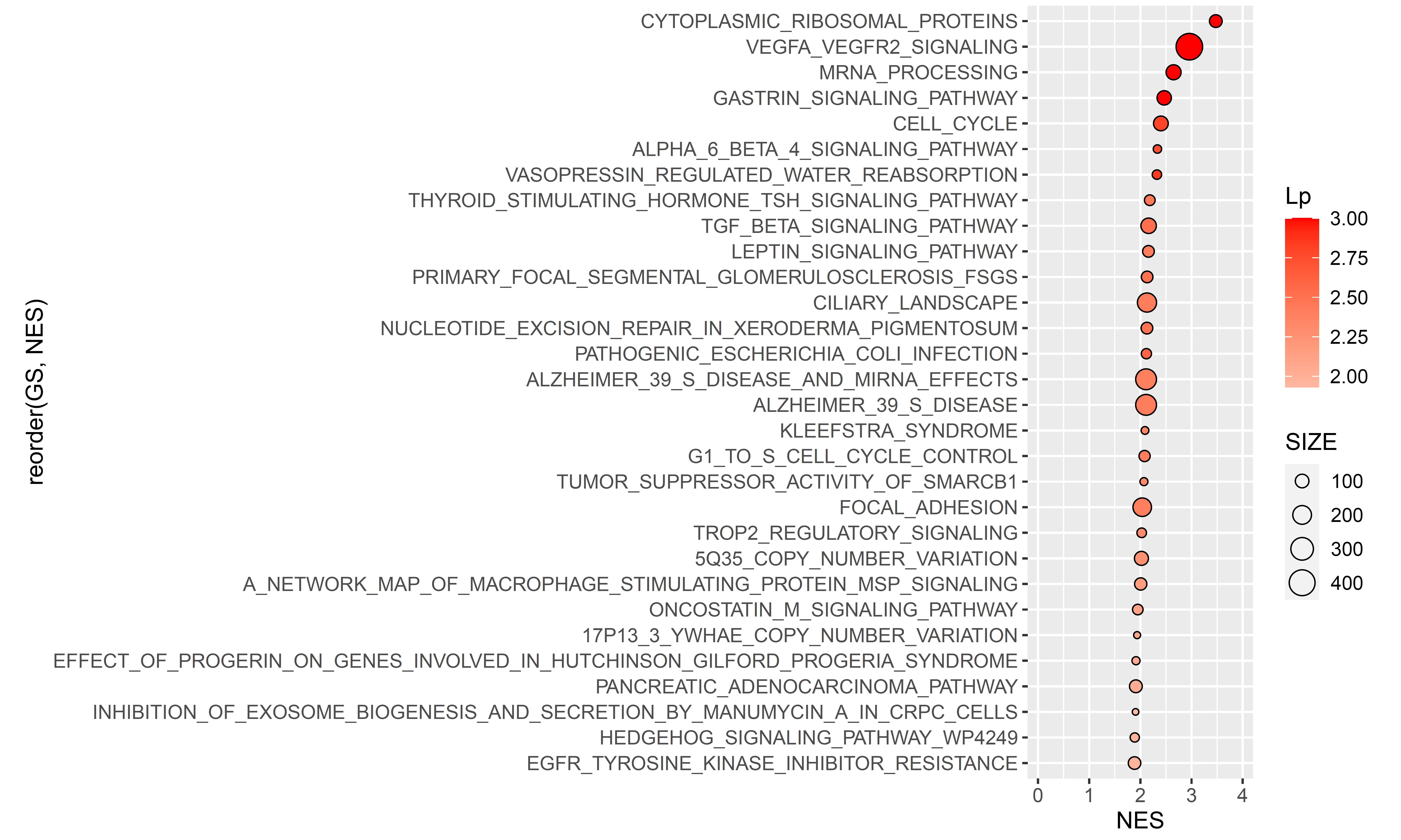
**

A

B

1/6G

1G

Sp7

RUNX2

BMP2


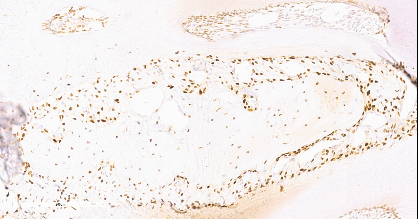

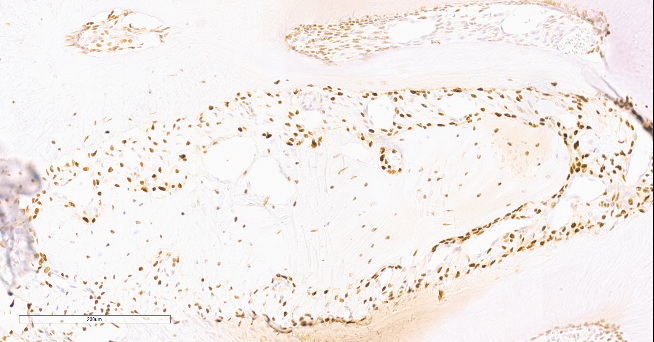

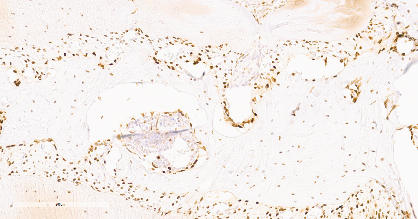

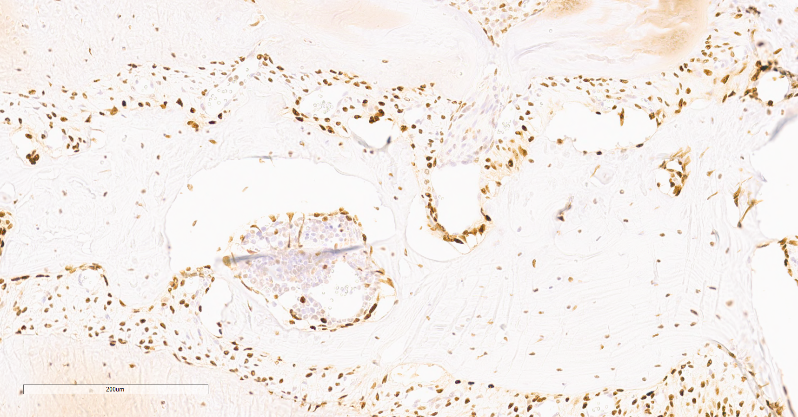

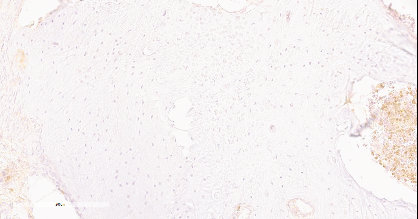

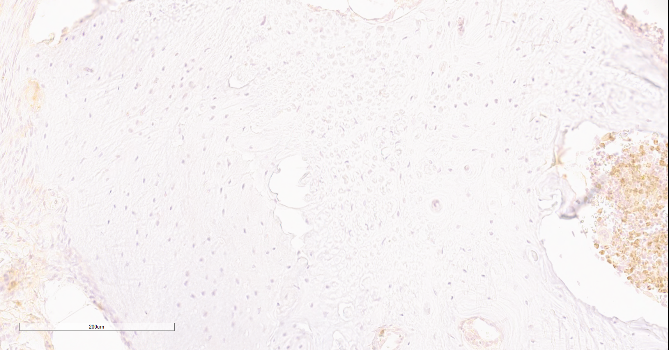

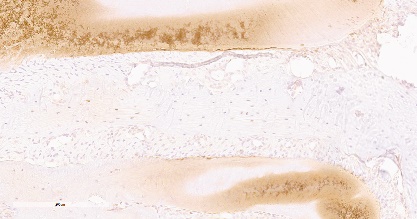

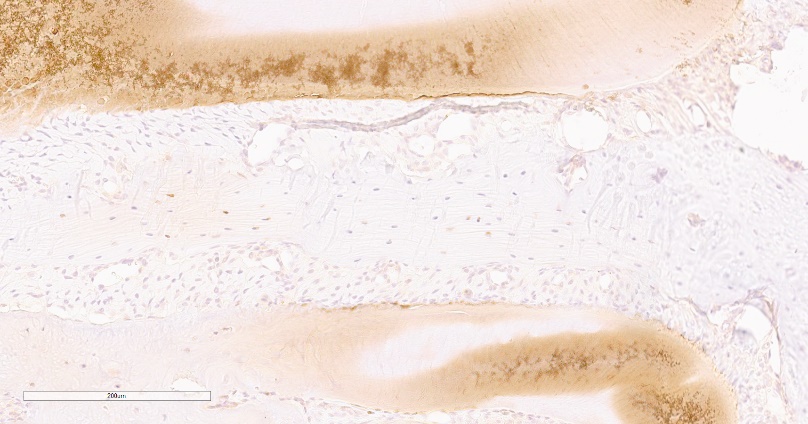

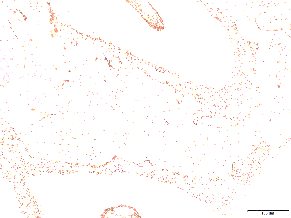

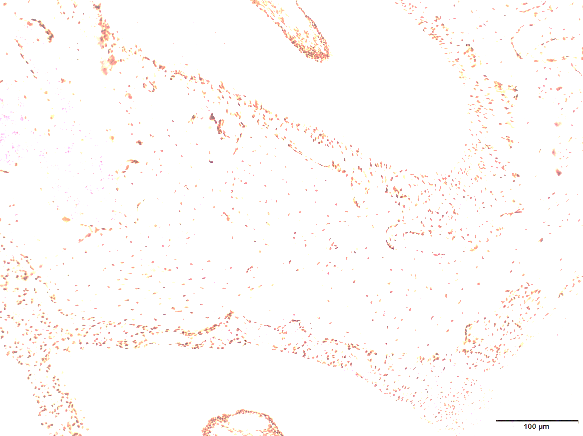

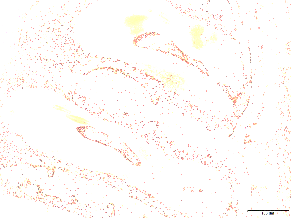

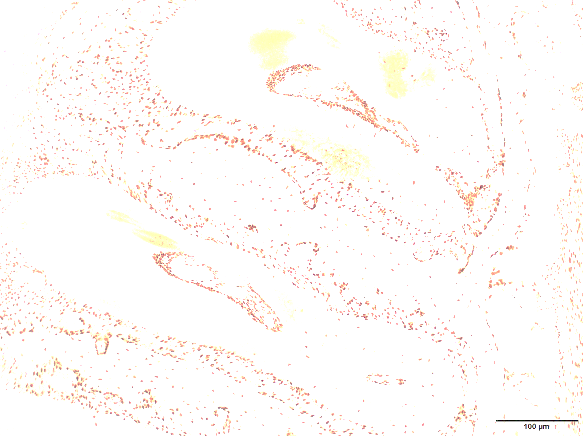


**Supplementary Fig. 1. Differences in bone maturation markers between the 1/6G and 1G groups.** (A) Gene Ontology analysis based on microarray data and gene set enrichment analysis. Headgehog and transforming growth factor-β (TGF-β) signaling were significantly promoted according to gene set enrichment analysis (GSEA). (B) The expression of RUNX family transcription factor 2 (RUNX2), bone morphogenetic protein 2 (BMP2), and Sp7 transcription factor (Sp7) did not significantly differ between the 1/6G and 1G groups based on histological findings and gene expression data. Scale bar = 200 µm. ns, Not significant.
